# Evaluating inter-study variability in phthalate and trace element analyses within the Children’s Health Exposure Analysis Resource (CHEAR) using multivariate control charts

**DOI:** 10.1038/s41370-021-00293-w

**Published:** 2021-02-18

**Authors:** Matthew J. Mazzella, Dana Boyd Barr, Kurunthachalam Kannan, Chitra Amarasiriwardena, Syam S. Andra, Chris Gennings

**Affiliations:** 1 Department of Environmental Medicine and Public Health, Icahn School of Medicine at Mount Sinai, New York, NY USA; 2 Department of Environmental Health, Rollins School of Public Health, Emory University, Atlanta, GA USA; 3 Department of Pediatrics and Department of Environmental Medicine, New York University School of Medicine, New York, NY USA

**Keywords:** Phthalates, Metals, Empirical/Statistical Models, Analytical Methods

## Abstract

**Background:**

The Children’s Health Exposure Analysis Resource (CHEAR) program allows researchers to expand their research goals by offering the assessment of environmental exposures in their previously collected biospecimens. Samples are analyzed in one of CHEAR’s network of six laboratory hubs with the ability to assess a wide array of environmental chemicals. The ability to assess inter-study variability is important for researchers who want to combine datasets across studies and laboratories.

**Objective:**

Herein we establish a process of evaluating inter-study variability for a given analytic method.

**Methods:**

Common quality control (QC) pools at two concentration levels (A and B) in urine were created within CHEAR for insertion into each batch of samples tested at a rate of three samples of each pool per 100 study samples. We assessed these QC pool results for seven phthalates analyzed for five CHEAR studies by three different lab hubs utilizing multivariate control charts to identify out-of-control runs or sets of samples associated with a given QC sample. We then tested the conditions that would lead to an out-of-control run by simulating outliers in an otherwise “in-control” set of 12 trace elements in blood QC samples (NIST SRM 955c).

**Results:**

When phthalates were assessed within study, we identified a single out-of-control run for two of the five studies. Combining QC results across lab hubs, all of the runs from these two studies were now in-control, while multiple runs from two other studies were pushed out-of-control. In our simulation study we found that 3–6 analytes with outlier values (5xSD) within a run would push that run out of control in 65–83% of simulations, respectively.

**Significance:**

We show how acceptable bounds of variability can be established for a given analytic method by evaluating QC materials across studies using multivariate control charts.

## Introduction

The Children’s Health Exposure Analysis Resource (CHEAR) program is a consortium that includes a network of laboratories and provides the opportunity for the children’s health research community to expand their research goals by offering assessment of a wide range of environmental chemicals utilizing a researcher’s existing biological specimens [1]. Resulting data, along with participant epidemiological data, are eventually compiled in a publicly accessible repository. In order to leverage the benefits of consortium programs such as CHEAR, it is important to have quality control (QC) systems available which allow researchers to assess inter-study and inter-laboratory variability allowing for the combination of data across studies, the benefits of which include increased sample sizes and improved generalizability [2].

The laboratory network within CHEAR includes six laboratory hubs (LHs). In order to maintain consistency across laboratories, each LH participates in multiple proficiency assessment schemes per year. In addition, CHEAR has distributed common QC materials, CHEAR QC pools, to each LH that are to be analyzed concurrently with study samples. These common QC materials allow for the assessment of variation within and between studies and LHs. Generally in CHEAR, when assessing a class of environmental exposure chemicals, the assigned LH runs a core set of analytes for that analytic method. Considering the fact that each laboratory is using different extraction methods, instrumental platforms and quantification methods, variability is expected to be inherent within and between assays. Nevertheless, it is important to assess if such variabilities are in- or out-of-control within and between assays.

Among various quality assurance and control protocols followed in CHEAR labs, two sets of QC pools were analyzed with every batch of samples. We selected data for seven phthalate metabolites in two sets of urine QC pools analyzed by three laboratories on five different studies to examine within and between LH variabilities. We demonstrate the use of multivariate control charts with Hotelling’s *T*^2^ as the charting statistic [3–5] to evaluate whether the values for the core set of analytes were in-control for each run within and across studies and LHs. Interpretation of out-of-control runs observed in this evaluation motivated us to address the question of how much variance in a single or multiple analyte(s) would it take to cause a run to be designated as out-of-control. We used QC pool data across 12 trace elements that were determined to be in-control to simulate out-of-control conditions to characterize use of the control charts.

## Methods

### QC pool creation (phthalates)

Ten liters urine (10 L) were collected from anonymous donors at Emory University using an Institutional Review Board-approved protocol for collecting urine for QC pools. Individual samples were pooled and mixed thoroughly. In addition, 500 mL urine was collected anonymously from smokers at the University of Minnesota and were shipped on dry ice, overnight to Emory University. Pool A consisted of the pooled urine from Emory. Pool B was created to mimic second-hand tobacco exposures by combining the 500 mL smokers’ urine with 4.5 L of Pool A. After mixing, 1.5 mL aliquots were pipetted into vials, labeled and frozen. The labels corresponded to typical CHEAR sample identification labels so that the samples were blinded to the analyst and that Pools A and B could not be differentiated. The key for the samples was provided to the CHEAR Data Center [1]. Each LH was shipped equal amounts of pools A and B, overnight on dry ice, and were instructed to have a sample coordinator (not the analyst) insert 3 of each pool into each grouping of 100 randomized samples for every organic chemical analysis in urine.

### Study design

#### Assessment of phthalate metabolites in urine

Internal controls were included for multiple studies in CHEAR to allow for intra- and inter-study and LH evaluations. For the analyses of phthalate metabolites in urine, the CHEAR QC pools A and B were evaluated across batches analyzed for multiple studies in three CHEAR LHs. There were between 4 and 33 aliquots of each CHEAR QC pool analyzed per study. Statistical analyses were conducted based on single run values. For these analyses we define a run as a set of analytic samples within a batch associated with a given QC sample, where there may be more than one run per batch. There were 7 common analytes within the phthalate metabolite method across these studies including mono-benzyl phthalate (MBZP), mono-2-ethyl-5-carboxypentyl phthalate (MECPP), mono-2-ethyl-5-hydroxyhexyl phthalate (MEHHP), mono-2-ethyl-5-oxohexyl phthalate (MEOHP), mono-ethyl phthalate (MEP), mono-iso-butyl phthalate (MIBP), and mono-n-butyl phthalate (MNBP). All phthalate concentrations were log10 transformed prior to evaluation of intra- and inter-study variability using multivariate control charts. Studies #1 and #2 were analyzed at Lab X, studies #3 & #4 were analyzed at Lab Y, and study #5 was analyzed by Lab Z.

#### Simulation study

A simulation study was conducted in order to characterize the sensitivity of multivariate control charts to identify outliers. To accomplish this we looked to simulate outliers in a real-world ‘in-control’ dataset with an adequate sample size and a sizeable number of components. We utilized data from a single CHEAR study from one LH evaluating trace elements in whole blood that fit these requirements to create a baseline dataset. Two NIST samples, SRM995c levels 2 and 3, were run over ten batches in this CHEAR study. There were three runs of each NIST level per batch; runs were averaged by batch for these analyses. A criterion was implemented to only include trace elements with an overall coefficient of variance (CV) less than 20%, resulting in an ‘in-control’ baseline dataset with 12 elements, including Mg, Mn, Cu, Zn, As, Se, Mo, Cd, Sn, Cs, Ba, and Pb. Measured concentrations of SRM at both levels were well beyond limits of detection (LOD) (greater than 5 × LOD) for all elements included in this dataset.

Simulated datasets were created by randomly selecting 1–6 of the 12 elements in a single batch of the baseline dataset for each NIST sample and increasing the batch average concentrations by 12.5%, 25%, 50%, 100%, 200%, or 400%. Values were then log10 transformed. One hundred datasets were created per batch for each manipulation condition and analyzed utilizing the multivariate control charts for a total of 1000 models tested for each manipulation condition. We calculated the percent of models that were out of control at each condition for both NIST samples combined.

We then created simulated datasets by utilizing a similar method, but instead of a fixed percent increase, the batch average concentrations of 1 to 6 elements were increased by multiples of the standard deviation of the log10 transformed values for a given element. Like the previous set of simulated datasets, 100 datasets were created per batch for each manipulation condition and analyzed utilizing the multivariate control charts for a total of 1000 models tested for each condition. The percent of models that were out of control at each condition for both NIST samples were calculated and plotted.

### Laboratory methods

#### Lab X phthalates

Urine samples were randomized prior to analysis to reduce the amount of analytic bias introduced into the data. A 1-mL aliquot of urine was spiked with isotopically labeled analogs of the target phthalate metabolites and subjected to an enzymatic hydrolysis to liberate glucuronide-bound conjugates. The hydrolysate was extracted using an ABS Elut-NEXUS solid-phase extraction (SPE) column, eluting with acetonitrile and ethyl acetate. The extract was concentrated to dryness and reconstituted in mobile phase for analysis using liquid chromatography-tandem mass spectrometry (LC–MS/MS). Analyte concentrations were calculated using an isotope-dilution method. LOD ranged from 0.1–0.5 ng/mL, typically. Two bench QC materials (one high and one low) and one blank sample were analyzed concurrently with each set of 28 samples. Further quality assurance measures were included in the sample analyses including NIST SRM 3672 and 3673 (one of each per 100 samples), CHEAR QC pools, and bi-annual participation in the German External Quality Assessment Scheme (G-EQUAS).

#### Lab Y phthalates

Lab Y followed the analytical method of the Centers for Disease Control and Prevention for phthalate metabolites in urine [6] with minor modifications [7]. Quantification was based on an isotope-dilution liquid chromatography and tandem mass spectrometry method [8]. In brief, ^13^C_4_- or D_4_-labeled internal standards were added to each sample, metabolites were treated with β-glucuronidase from *Escherichia coli*-K12 (product # 3707601001, Roche Diagnostics through Sigma Aldrich), followed by SPE with an Oasis HLB hydrophilic-lipophilic balanced reversed-phase 96-well plate (30 mg sorbent per well, 30 µm particle size; Waters Corporation, Milford, MA). The procedure was automated using a liquid handler (epMotion 5075vtc; Eppendorf, Hauppauge, NY). The LC–MS/MS (UHPLC Nexera XR, Shimadzu and Sciex 6500 triple quadruple MS, AB Sciex; Framingham, MA) was operated in electrospray negative mode for ionization and multiple reaction monitoring for quantification. Chromatographic separation was achieved on a Kinetex biphenyl, 2.6 µm, 50 × 2.1 mm analytical column with 2 × 2.1 mm guard cartridge (Phenomenex Inc., Torrance, CA) using a mobile phase gradient with 0.1% acetic acid in LC–MS grade water and acetonitrile, respectively. The LOD for phthalate metabolites ranged from 0.05–0.50 ng/mL. As with Lab X, in addition to CHEAR QC pools A and B, Lab Y included NIST SRM 3672 for study #3 (three per 100 study samples), NIST SRM 3673 for both studies #3 and #4 (three per 100 study samples), and participates bi-annually in the German External Quality Assessment Scheme (G-EQUAS).

#### Lab Z phthalates

Lab Z used methods similar to those of the other two labs. Quantitative detection of phthalate metabolites was achieved utilizing a solid-phase extraction (SPE) method followed by enzymatic deconjugation of the glucuronidated phthalate monoesters coupled with HPLC-ESI-MS/MS, as previously described [9]. Assay precision is improved by incorporating ^13^C_4_- or D_4_-isotopically labeled internal standards for each of the phthalate metabolites. This selective method allowed for rapid detection of metabolites of phthalates with the majority of LOD in the range of 0.01–0.20 ng/mL (Table S1). Quality assurance measures included the insertion of CHEAR QC pools A and B, NIST SRM 3672 and 3673 (one per 100 samples), and bi-annual participation in the German External Quality Assessment Scheme (G-EQUAS).

#### Trace element analysis in blood

In brief, blood samples (200 µl) were diluted with a diluent solution (8.8 ml containing 0.5 % HNO_3_, 0.005% Triton X-100, mixed internal standard) in a 15 ml polypropylene trace metal-free Falcon tubes (VWR^®^ Metal-Free Centrifuge Tubes). All sample preparation was performed in an ISO Cass 5 laminar flow clean hood in the ISO Class 6 clean room. Analytes were quantified from matrix matched calibration standards using Agilent 8800 ICP Triple Quad (ICP-QQQ) (Agilent technologies, Inc., Wilmington, Delaware, USA) MS/MS with appropriate cell gases to eliminate molecular ion interferences. Internal standards (yttrium, indium, tellurium and lutetium) were used to correct for the differences sample introduction, ionization and reaction rates in the reaction cell. All runs included 5% QC samples, blinded prior to receipt in the lab, including duplicates, field blanks and NIST SRM 955c (Toxic elements in Caprine Blood, Gaithersburg, MD). QA/QC procedures included analyses of initial calibration verification standards and continuous calibration verification standards (CCVS) mixed element standards at two different concentration levels, procedural blanks, duplicates and in-house-pooled blood sample (IHB) to monitor the accuracy and reproducibility of the analysis. CCVS and IHB were run after analysis of every ten samples. All lab recovery rates for QC standards and spiked samples were 85–115% and precision (given as %RSD) was <10% for samples with concentrations >LOQ. The LOD for analytes were between 0.02 and 2 ng/mL and LOQ ranged between 0.07 and 6.4 ng/mL.

### Statistical methods

#### Summary statistics for phthalate metabolites in urine

Means and percent CV were calculated for each phthalate per study and overall. Spearman rank correlation coefficients were calculated for each study.

#### Multivariate control charts

We assume that repeated evaluations of the QC pools (used for inter-study evaluation of phthalates) or NIST standards (used as basis for simulation study) are generally “in-control” so that the population means and covariance structures can be estimated by sample statistics. To set notation, following Tracy et al. [3], assume *n* samples of *p* components comprise a *p*-variate vector $${\mathbf{X}}_i = \left[ {\begin{array}{*{20}{c}} {X_{i1}} \\ \vdots \\ {X_{ip}} \end{array}} \right]$$ with estimated mean vector $${\bar{\mathbf{X}}} = \left[ {\begin{array}{*{20}{c}} {\bar X_1} \\ \vdots \\ {\bar X_p} \end{array}} \right]$$ and estimated covariance matrix ${\mathbf{S}} = \frac{1}{{n - 1}}\mathop {\sum}\nolimits_{i = 1}^n {\left( {{\mathbf{X}}_i - {\bar{\mathbf{{X}}}}} \right)} \left( {{\mathbf{X}}_i - {\bar{\mathbf{{X}}}}} \right)^\prime$. The Hotelling’s *T*^2^ statistic charting statistic is defined as $Q_i = \left( {{\mathbf{X}}_i - {\bar{\mathbf{{X}}}}} \right)^\prime {\mathbf{S}}^{ - 1}\left( {{\mathbf{X}}_i - {\bar{\mathbf{{X}}}}} \right)$. Conveniently, the T^2^ statistic is equivalent to the sum of standardized principal components (PCs) of the covariance matrix for each QC pool or NIST standards. However, with *p* large and *n* small, we approximate *T*^2^ using a subset (S) of the PCs; thus, *T*^2^ is approximated as $\mathop {\sum}\nolimits_{j = 1}^S {\left( {\frac{{\rm{PC}}_{j}}{{\rm{SE}(\rm{PC}_{j})}}} \right)^2}$. Control limits can be calculated when the number of observations is greater than the number of components in the statistic plus two. Upper and lower limits were calculated using the 99% confidence interval from the distribution of the *T*^2^ statistic for the inter-study analyses and simulation study.

Statistical analyses were conducted with SAS 9.4 (Cary, NC). A template for SAS code is provided in supplemental material (Template S1).

## Results

### Variability assessment of phthalate metabolites in urine

The summary statistics for the seven common phthalates in the CHEAR QC urine pool over five CHEAR studies are shown in Table 1 (source data in Table S2). For both CHEAR QC pools A and B, 4 of the 7 phthalates, MECPP, MEHHP, MEP, and MIBP, had an overall CV greater than 20. Looking at intra-study precision, study #1 showed a single phthalate, MIBP, with a CV above 50% in both QC pools. Study #3 showed 2 of the 7 phthalates with a CV above 20%, MBZP and MEOHP, but for QC pool A only. For study #5, MBZP and MECPP had CVs above 20% in both QC pools, while MEHHP had a CV above 20% for QC pool B only.

**Table 1:** Summary statistics for CHEAR QC pools A and B by study showing mean values for each of the seven common phthalates by study and overall and the intra- and inter-study percent CVs.

		Overall		Study #									
		(*N* = 90)		1 (*N* = 4)		2 (*N* = 25)		3 (*N* = 7)		4 (*N* = 33)		5 (*N* = 21)	
		Mean	%CV	Mean	%CV	Mean	%CV	Mean	%CV	Mean	%CV	Mean	%CV
Pool	Analyte												
A	MBZP	1.00	16	0.84	11	0.99	8	0.98	27	1.02	12	1.02	22
	MECPP	4.71	39	5.13	1	7.28	3	3.91	15	4.28	9	2.50	30
	MEHHP	2.89	25	2.77	4	2.92	6	2.19	11	2.36	11	3.94	17
	MEOHP	2.13	20	1.75	3	2.04	5	2.47	22	2.47	13	1.67	18
	MEP	16.0	21	13.50	13	15.31	4	13.07	9	13.82	9	21.80	1
	MIBP	3.82	23	2.60	51	2.88	16	3.92	14	4.31	11	4.38	13
	MNBP	7.86	20	5.41	11	6.17	6	7.93	13	8.26	10	9.70	6
B	MBZP	1.41	16	1.26	6	1.39	4	1.59	12	1.52	11	1.23	24
	MECPP	6.05	38	6.28	2	9.05	4	5.26	14	5.91	9	2.95	31
	MEHHP	4.34	29	4.24	4	4.18	8	3.17	13	3.54	7	6.19	21
	MEOHP	2.76	18	2.42	5	2.63	2	3.27	7	3.18	11	2.15	16
	MEP	16.9	21	13.33	14	16.42	5	13.29	11	14.57	7	22.87	1
	MIBP	4.60	26	5.93	62	3.51	19	4.69	11	4.91	12	5.13	17
	MNBP	8.26	18	6.93	13	6.36	5	8.25	7	8.89	10	9.79	6

**Fig. 1:**
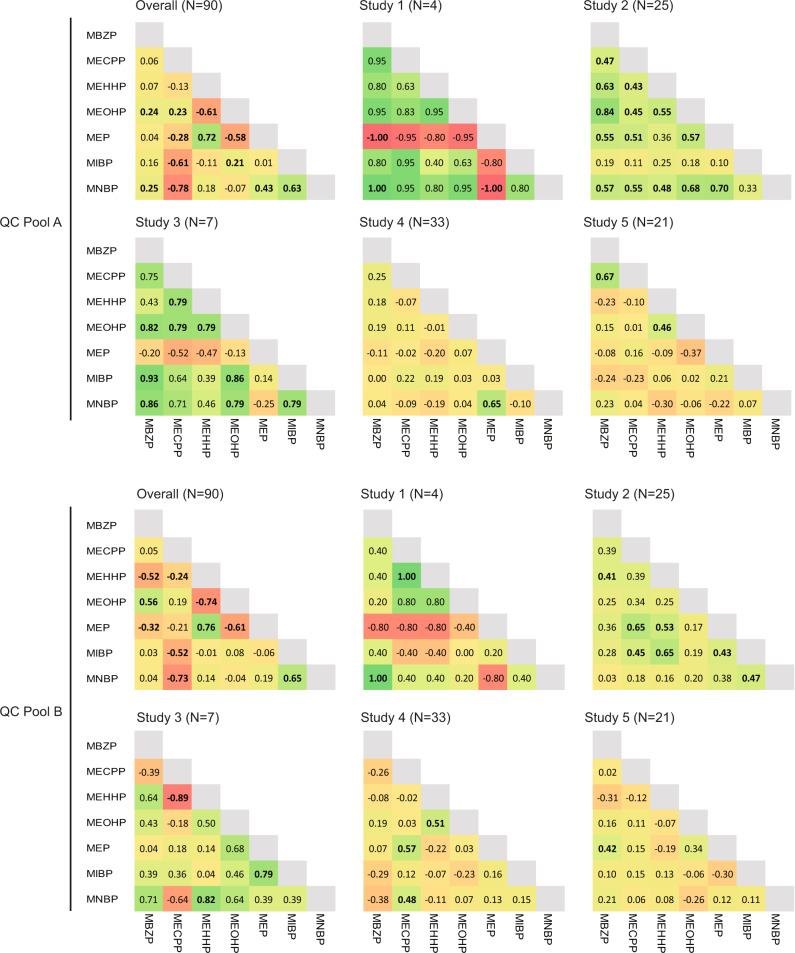
Spearman correlation matrices for the 7 common phthalates by QC pool for each of the 5 CHEAR studies and overall. The three color scale was used to indicate a correlation coefficients approaching −1 (red), 0 (yellow), or 1 (green); significant correlation coefficients in bold (color figure online).

Looking at the Spearman correlation coefficients among the phthalates by study, there were stronger correlations between analytes for both CHEAR QC pools in studies #1 [range: Pool A: −1.0, 1.0, Pool B: −0.8, 1.0] and #3 [range: Pool A: −0.54, 0.93, Pool B: −0.89, 0.82], than in studies #2 [range: Pool A: 0.09, 0.84 Pool B: 0.01, 0.65], #4 [range: Pool A: −0.20, 0.65 Pool B: −0.31, 0.42], and #5 [range: Pool A: −0.37, 0.67 Pool B: −0.38, 0.57] (Fig. 1). The sample sizes were smaller in studies #1 and #3 (*N* = 4 and 7, respectively) compared to studies #2, #4, and #5 (*N* = 25, 33, and 21, respectively), making the comparison somewhat tenuous. However, the point is that comparison of measures of QC pools involves point estimates of means and standard deviations, but should also accommodate comparisons of bivariate correlation and covariance patterns.

**Fig. 2:**
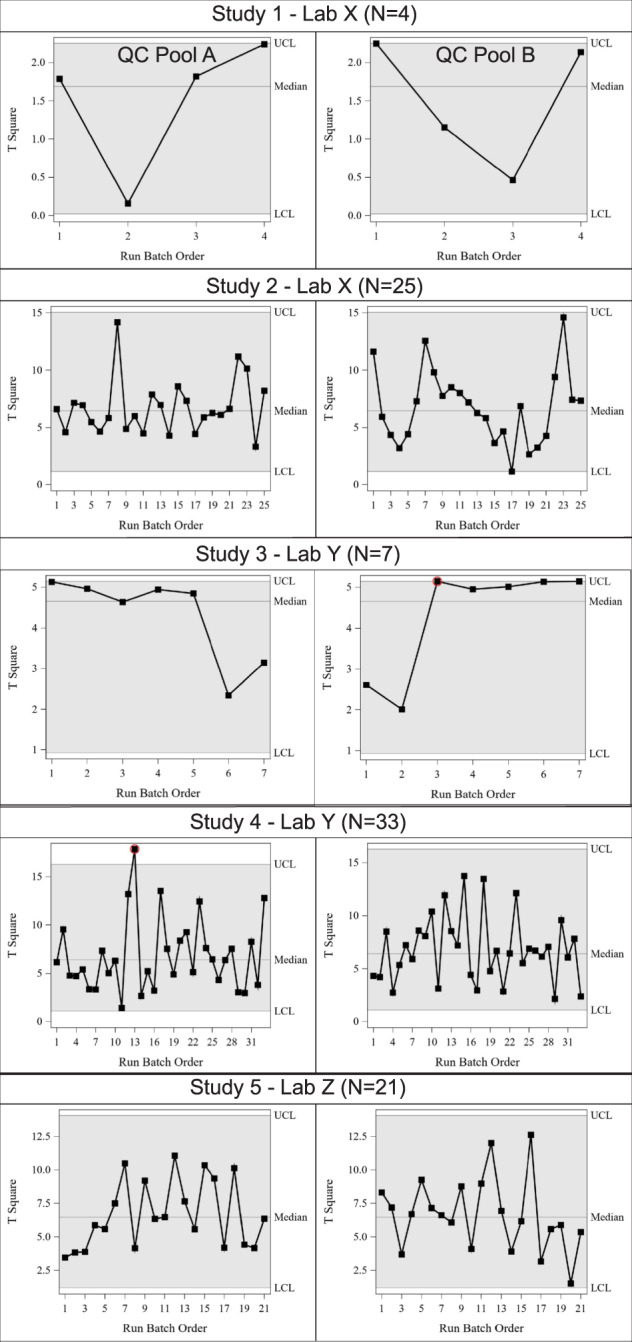
Multivariate control charts for 7 phthalates from CHEAR QC pools A (left panel) & B (right panel) from 5 CHEAR studies assessed by 3 different CHEAR lab hubs. The control charts show the *T*^2^ statistic plotted by run order for each study. The area shaded in gray represents the in control range with reference lines for the median, upper confidence limit (UCL), and lower confidence limit (LCL).

In order to examine the variance and covariance of the phthalates in aggregate, the *T*^2^ statistic was calculated for each run by study. The *T*^2^ statistic was calculated from the sum of two PCs for study #1, accounting for 95% of the total variability, 7 PCs for study #2 (100% of variability), 5 PCs for study #3 (100% of variability), 7 PCs for study #4 (100% of variability), and 7 PCs for study #5 (100% of variability). When calculated separately by study, the multivariate control charts show that each run within studies #1, #2, and #5 is in control for both QC pools, i.e., the *T*^2^ for each run is below the upper confidence limit (UCL), while a single run is out-of-control in study #3 for QC pool B and in study #4 for QC pool A (Fig. 2). A run is “out-of-control” if the *T*^2^ for that run falls above the UCL. The gray area in the figures represents the middle 99% of the distribution of the test statistic. Note the distribution is more skewed when the sample size is small.

**Fig. 3:**
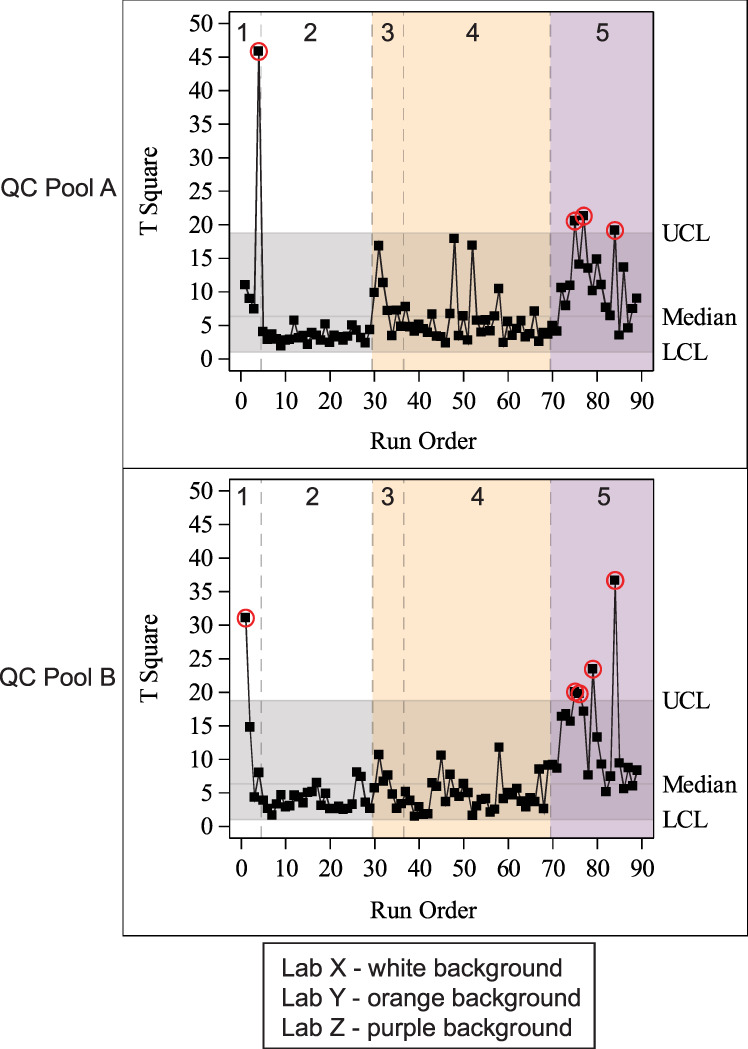
Multivariate control charts for 7 phthalates run for the CHEAR QC pools A (top panel) & B (bottom panel) from a combination of five CHEAR studies assessed by three different CHEAR lab hubs. The control charts show the *T*^2^ statistic plotted by run order for each study. The area shaded in gray represents the in control range with reference lines for the median, upper confidence limit (UCL) and lower confidence limit (LCL). Batches from separate studies are separated by a vertical dotted line and labeled with the study number. White plot background indicates a study analyzed by Lab Y (studies 1 and 2), the orange indicates a study analyzed by Lab Y (studies 3 and 4), and purple Lab Z (study 5). Out of control runs are circled in red (color figure online).

To allow for both inter-lab and inter-study comparisons, the *T*^2^ statistics per run were recalculated to include the data of the seven common phthalates in CHEAR QC pools A and B from all five studies, thereby resetting the confidence intervals for the control charts. The *T*^2^ statistic was calculated from the sum of 7 PCs for the 5 combined studies, accounting for 100% of the variability. The combination of these sets of data readjusted the mean, variance, and covariance estimates such that all runs in studies #3 and #4 are now in control, while, for both QC pools, a single run in study #1 and multiple runs in study #5 are now out of control (Fig. 3).

**Fig. 4:**
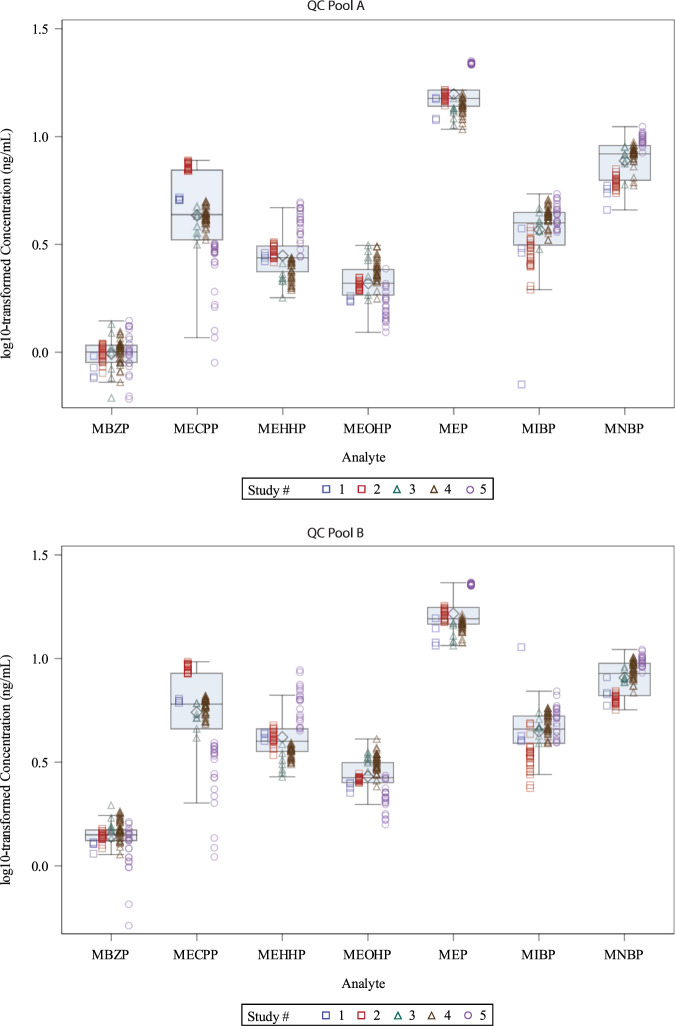
Plot of log10 transformed phthalate concentrations from QC pools A (top panel) and B (bottom panel) run for the 5 CHEAR studies with overlaid box plot analysis to indicate outlier values. Square, triangular, and circular markers indicate analyses run by Labs X, Y, and Z, respectively.

We then examined the five study *T*^2^ statistic contribution plots (Figs. S1 and S2) for the out-of-control runs from study #1, runs 4 and 1 for pools A and B, respectively. For both runs, MIBP was the largest contributing phthalate to this value as identified based on contribution plots. Follow-up examination of z-scores for the combined dataset (Table S3) revealed MIBP to be 3.6 standard deviations below the mean in run 4 for QC pool A and 5.8 standard deviations above the mean in run 1 of QC pool B. An outlier MIBP value for a single run in both QC pools for study #1 can also be seen in the box plots of the log10 transformed values in Fig. 4.

### Simulation study using 12 trace elements

**Fig. 5:**
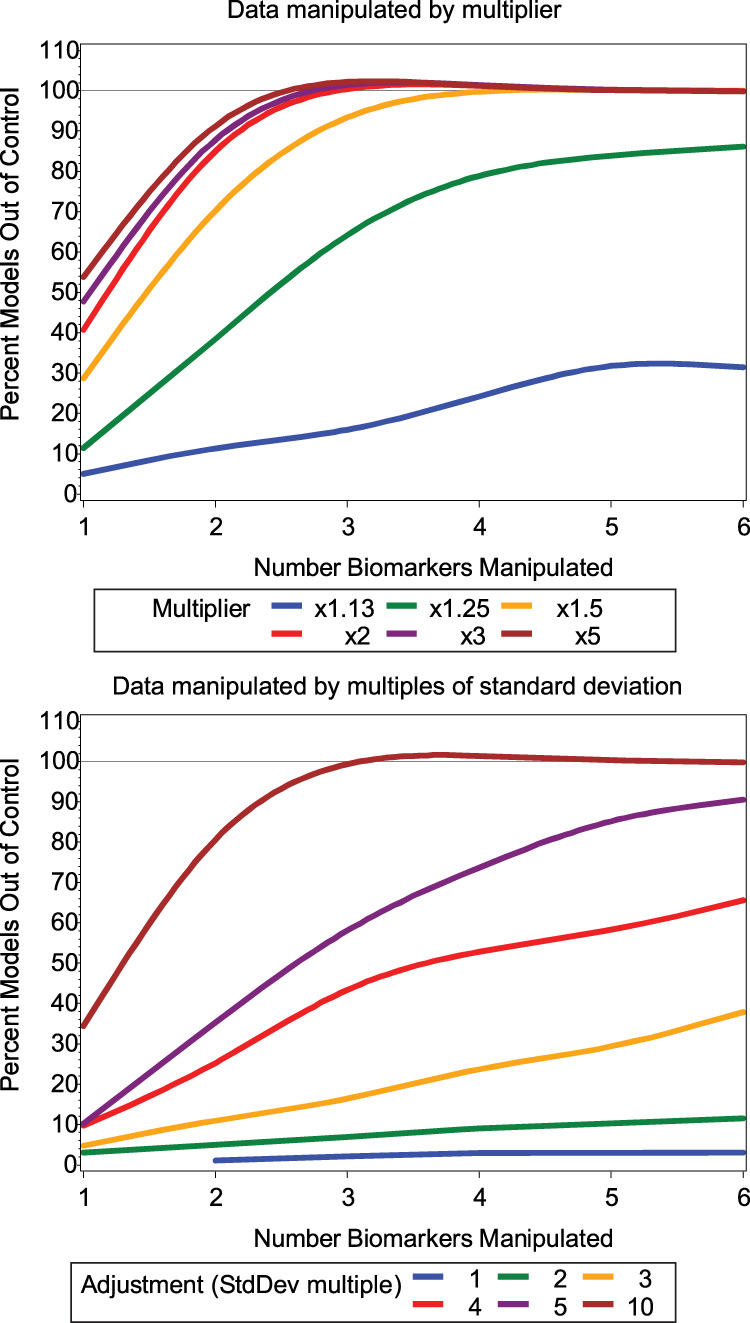
Graph of multivariate control chart response to varying degrees of data manipulation shown as a percent of models driven “out-of-control” for each simulated condition. For each model in this simulation, 1–6 analyte measures in a single batch were increased by a multiplier (1.13–5; top panel) or multiples of a given analyte’s standard deviation (1–10; bottom panel) in an otherwise “in control” dataset of 12 trace elements in whole blood from NIST SRM 955c level 2 and level 3 samples run over ten batches. Two thousand models were tested per condition, one hundred per sample per batch.

In order to determine how much variance in a single or multiple analyte(s) it would take to cause a run to be designated as out-of-control, we created simulation datasets from a baseline in-control dataset of 12 trace elements run over 10 batches, utilizing batch average values of the runs (Fig. S3). We augmented the batch average value of 1–6 randomly selected elements by a set of multipliers, increasing these batch average values by 13% up to 400% (Fig. 5) and counted the percent of 2000 scenarios, 1000 per NIST sample, resulting in *T*^2^ statistics that exceeded the UCL. For example, when a random batch average value of a single element out of 12 in this otherwise in-control dataset was doubled, the batch was out of control in about 40% of simulated models. Augmented batches were out-of-control in all of the simulated models when a third of the elements (4 out of 12) were doubled. To test a subtler shift, the batch average values for half of the elements (6 out of 12) in a single random batch were increased by 25%, resulting in an out-of-control batch in roughly 90% of the simulated models.

Since the effect of the percent increase in a batch average value would be influenced by the variance of a selected analyte, we also simulated datasets by augmenting the batch average values of the same in control baseline dataset by multiples of the standard deviation for a given element (Fig. 5). The results of this simulation estimated, for example, that if the batch average value for a third of the elements (4 out of 12) within a batch were outliers (~average + 4xSD), then that batch would be out-of-control in roughly half of the models.

## Discussion

We have demonstrated the utility of using multivariate control charts for the CHEAR QC pools A and B in different contexts, i.e., evaluating intra-study data, and inter-study, inter-lab data. The charting statistic identifies batch runs that are outliers and the contribution plots demonstrate which component(s) most contribute to the extreme statistic [10, 11]. Through simulation, we characterized the degree (in units of percentage and standard deviations) to which a batch would be altered to change the set to be determined out of control.

It is imperative in consortia programs, such as CHEAR which has multiple labs, that a tool is available that allows one to track intra- and inter-laboratory measures and evaluate the consistency required to combine study data. For example, consider the scenario where environmental exposure data from two studies measured from two labs are to be combined. Differences in exposure concentrations should be due to true exposure differences and not laboratory bias. The insertion of the common QC pools by all of the participating CHEAR LHs for every run within each study provides the opportunity to track consistency over time, study, and laboratory, using statistical tools such as multivariate control charts. Other QC materials can be used for these purposes, such as NIST SRMs, but there is a cost consideration that may limit the ability to insert at the appropriate rates. Within the CHEAR program, as an example, the common QC pools are inserted by LHs at a rate of 3 samples of each pool per 100 study samples. These charts from QC materials permit the visualization of data that accommodates differences in means and standard deviations across analytes and complex covariance patterns between analytes for a given analytic method.

Multivariate control charts can be applied to determine if all runs, across datasets considered for combination, are in-control. The identification of out-of-control runs within a study could trigger follow-up investigation to the cause of the variation. As the simulation study demonstrated, a single analyte may be the main contributor to the variability. In this case, that one analyte can be omitted from the combined dataset. Alternatively, there may be a single run or batch in one of the studies that is highly variable and can be omitted. Or it can be determined that the differences in the QC results are such that the datasets should not be combined.

The practice of using study-wide coefficients of variance to evaluate “in-control” conditions is limited. A given quality objective for precision, e.g., %CV< 20, may not always be relevant for certain analytes in an analytic method whose measurements can be highly variable, or if the measured values in a common pool are approaching the LOD for an analyte [12, 13]. In addition, CVs alone can indicate changes in variance but not whether the covariance between analytes in a given analytic method for a specific run is consistent with the overall covariance structure. The ability to evaluate both the variance and covariance using the *T*^2^ statistic is advantageous in these evaluations.

In addition, the approximation of the *T*^2^ statistic using a subset of PCs permits the advantage of using it as a charting statistic for the case where the number of components (*p*) greatly exceeds the sample size (*n*)—i.e., the *p* > *n* case [14]. The CHEAR Data Center is proposing the use of the charting statistic in QC evaluations for more expansive analytic methods such as in metabolomics where there may be tens of thousands of components.

There are currently limited studies with overlapping analytic methods, leading to a sample size too small to establish reliable estimates of the true means and covariance structure for each of the CHEAR QC pools. Furthermore, since the true mean and covariance structure is not established and the number of QC samples run within each study varies, the *T*^2^ statistics are biased toward the study with the largest *N*, study #4 in the case of phthalates. These issues will be mitigated when more studies are included in the analyses resulting in a larger overall sample size. In the long term, the historical data will provide improved estimates of the mean and covariance structure with negligible change in estimates with additional data.

An additional limitation to this approach is that it relies on the presence of a consistent common core of exposure analytes for a given method. For example, in our phthalates analyses, Labs X and Y each measured nine phthalates and Lab Z measured twenty phthalates. There was a common set of seven phthalates amongst these studies which determined the common core of exposure analytes. This may not always be feasible. The optimal core set of exposure analytes may vary over time, such as when replacement chemicals are included in a method. The process may require being “re-initiated” with changes in the core set for a given method. It also notable that the QC urine pools contained concentrations of 7 phthalate metabolites below the national average and therefore expected to result in greater variability at such low levels of analysis.

## Conclusions

For the CHEAR program, as well as similar consortia programs, it is essential to develop systems that allow for the assessment of consistency over time and with differing laboratories, allowing for the combination of datasets. With the evaluation of QC materials common across studies by all participating LHs within our consortia and the utilization of multivariate control charts to evaluate the results from these QC materials over time, we have established a system that can both be used for intra-study QC, identifying runs requiring further examination or re-analysis, and inter-study evaluation of fitness for combinability.

## Supplemental Information

Supplemental Information Table S2 Table S3
