# Supplementary material for: Evaluating inter-study variability in phthalate and trace element analyses within the Children’s Health Exposure Analysis Resource (CHEAR) using multivariate control charts
Source: J Expo Sci Environ Epidemiol. 2021 Feb 18;31(2):318–27. doi: 10.1038/s41370-021-00293-w (PMC7952263; doi:10.1038/s41370-021-00293-w)

Supplemental Information

Template S1. Template for SAS code used to create multivariate charts for QC evaluations.

```
*MVCC macro; * data required in wide format;
%macro      MVCC  (dataset, analytes, components, pool);

      proc mvpmodel     data=&dataset
                        ncomp=&components
                        plots=all
                        out=outqc
                        outloadings=qcloadings;
                        where pool="&pool";
                        var &analytes;
                        run;

      proc mvpmonitor   history=outqc
                        loadings=qcloadings;
                        where pool="&pool";
                        time run;
                        tsquarechart/alpha=0.01 contributions outtable=temp;
                        label run='Run Order';
                        run;

%mend MVCC;
```

Table S1. Study specific LODs for the 7 common phthalates measured in CHEAR QC pools A and B from 5 CHEAR studies. All values are reported in ng/mL.

	**LOD**				
	**Study #**				
	**1**	**2**	**3**	**4**	**5**
**Analyte**					
**MBZP**	0.20	0.20	0.04	0.15	0.02
**MECPP**	5.00	2.10	0.06	0.15	0.02
**MEHHP**	0.40	0.40	0.07	0.15	0.20
**MEOHP**	0.40	0.40	0.04	0.15	0.01
**MEP**	0.40	0.40	0.18	0.15	0.10
**MIBP**	0.50	1.00	0.25	0.50	0.01
**MNBP**	0.50	0.20	0.39	0.50	0.20

Figure S1. Example of the T-square contribution plots generated from the five study inter-lab multivariate control chart for the seven common phthalates in pool A and B. This plot shows the individual variable contributions to the T square statistic for pool A, run #4 from study #1 and for pool B run #1 from study #1, both of which were determined to be out of control.

QC Pool A	QC Pool B

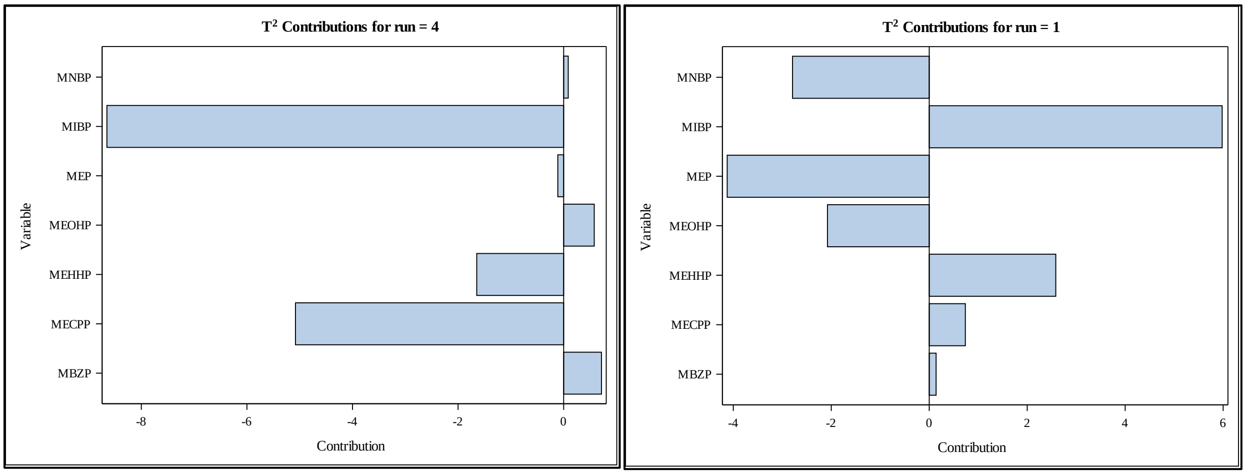

Figure S2. T-square contribution plots for out of control runs in study #5, generated from the five study inter-lab multivariate control chart for the seven common phthalates in pool A and B. This plot shows the individual variable contributions to the T-square statistic for runs #76, #78, and #85 of pool A and runs #76, #77, #80, and #85 of pool B, all of which were determined to be out of control.

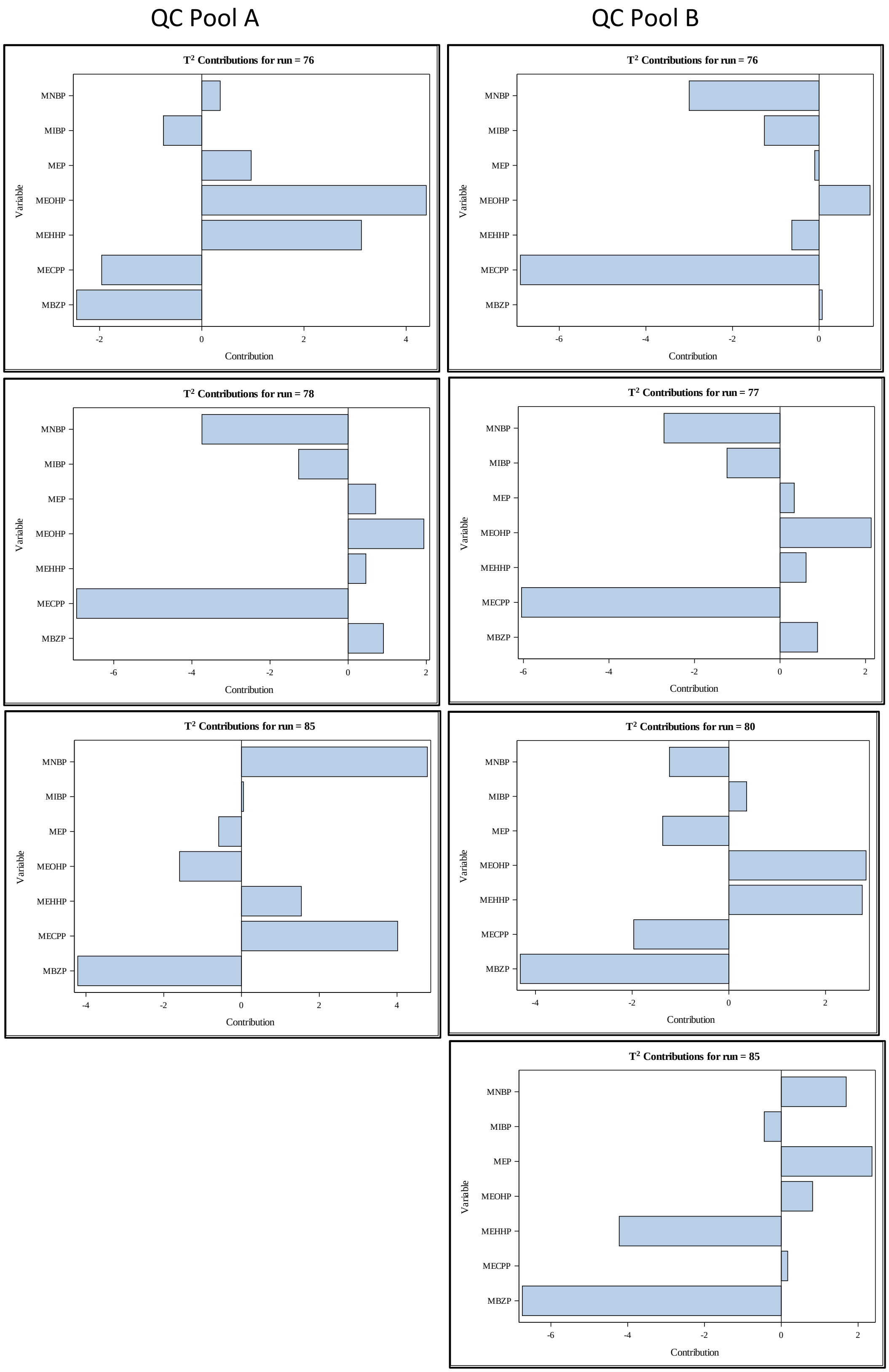

Figure S3. Multi-variate control charts for twelve metals in whole blood run for QC samples NIST SRM 955c level 2 (left) & level 3 (right) run in conjunction with a CHEAR study. The control charts show the T2 statistic plotted by batch order. The area shaded in grey represents the in control range with reference lines for the median, upper confidence limit (UCL) and lower confidence limit (LCL).

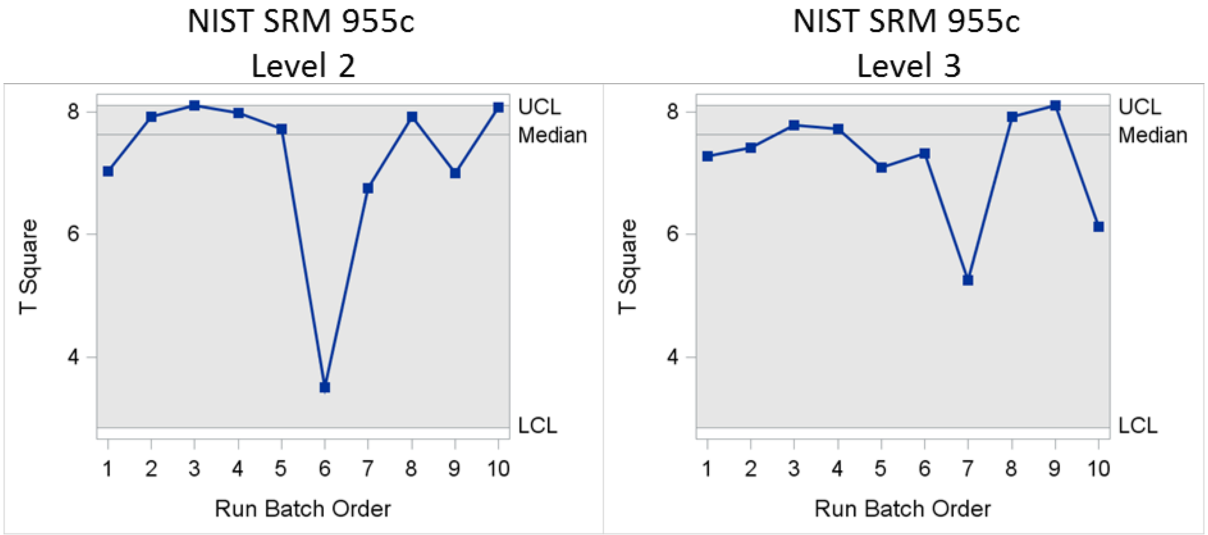

Supplement: Supplementary file 1 — Supplemental Information [file 41370_2021_293_MOESM1_ESM.pdf]
