# Supplementary material for: Evaluating inter-study variability in phthalate and trace element analyses within the Children’s Health Exposure Analysis Resource (CHEAR) using multivariate control charts
Source: J Expo Sci Environ Epidemiol. 2021 Feb 18;31(2):318–27. doi: 10.1038/s41370-021-00293-w (PMC7952263; doi:10.1038/s41370-021-00293-w)
Supplement: Supplementary file 2 — Table S2 [file 41370_2021_293_MOESM2_ESM.pdf]

Table S2. Values from the seven common phthalates measured in CHEAR QC pools A and B from 5 CHEAR studies. Batch numbers are provided per study and overall. All values are reported in ng/mL.

		Pool													
		A							B						
Study #	Run order	MBZP	MECPP	MEHHP	MEOHP	MEP	MIBP	MNBP	MBZP	MECPP	MEHHP	MEOHP	MEP	MIBP	MNBP
1	1	0.96	5.23	2.89	1.82	11.9	3.75	5.92	1.31	6.27	4.17	2.39	12.0	11.40	8.16
	2	0.85	5.10	2.80	1.75	12.1	2.89	5.72	1.29	6.43	4.42	2.50	11.6	4.01	6.86
	3	0.77	5.10	2.64	1.72	14.9	3.04	5.45	1.28	6.28	4.36	2.53	14.0	4.25	6.74
	4	0.76	5.07	2.74	1.72	15.1	0.71	4.57	1.15	6.13	4.02	2.26	15.7	4.06	5.95
2	5	1.03	7.40	3.10	2.21	15.9	3.65	6.85	1.36	8.49	4.19	2.53	15.4	4.87	6.55
	6	1.05	7.29	3.14	2.15	15.1	3.04	6.59	1.35	8.55	4.10	2.66	15.4	3.17	6.65
	7	1.09	7.19	3.19	2.22	15.6	2.55	6.24	1.38	8.52	4.02	2.66	15.6	3.43	6.65
	8	1.09	7.10	2.91	2.21	15.6	3.30	6.27	1.41	8.56	3.87	2.63	15.7	2.94	6.39
	9	0.94	7.07	2.61	1.99	14.8	2.96	6.12	1.36	8.56	4.04	2.53	15.5	2.77	6.17
	10	0.97	7.08	2.81	2.09	15.5	2.52	5.92	1.42	9.16	3.70	2.69	15.1	3.13	6.14
	11	1.00	6.96	2.92	1.99	15.2	3.36	6.04	1.36	9.25	3.69	2.80	16.3	3.39	6.30
	12	1.00	6.92	3.09	1.95	14.7	2.03	6.05	1.36	8.80	3.43	2.53	16.1	3.07	6.37
	13	0.98	7.54	2.96	2.00	16.1	2.49	6.20	1.45	8.53	4.47	2.61	16.4	2.85	6.07
	14	1.08	7.57	2.82	2.12	16.1	2.53	6.35	1.43	8.54	4.60	2.64	16.2	3.74	6.08
	15	0.98	7.48	2.74	2.05	15.4	2.76	6.42	1.52	9.69	4.79	2.68	17.1	3.56	6.19
	16	1.10	7.75	3.11	2.09	16.4	3.08	7.08	1.40	9.34	4.67	2.69	17.0	4.90	7.00
	17	1.06	7.62	3.24	2.05	16.4	3.13	6.62	1.41	9.46	4.42	2.60	17.4	4.78	6.30
	18	1.03	7.25	2.99	2.00	14.8	3.14	5.94	1.43	9.39	4.33	2.67	17.7	3.55	6.89
	19	0.97	7.36	2.97	1.97	14.5	3.80	5.93	1.40	9.45	4.59	2.65	18.0	4.34	6.59
	20	1.04	7.19	2.73	2.02	14.6	2.72	5.86	1.38	9.29	4.41	2.67	17.0	3.50	6.76
	21	1.07	7.47	3.09	2.13	16.0	3.20	6.16	1.38	9.19	4.20	2.61	16.2	3.32	6.17
	22	1.06	7.66	3.18	2.15	15.3	2.83	6.32	1.36	9.15	4.24	2.64	15.2	3.77	6.08
	23	0.90	7.53	2.74	1.93	14.9	2.66	5.80	1.41	9.41	4.27	2.59	16.9	3.78	6.56
	24	0.86	7.09	2.82	1.93	15.0	2.76	5.65	1.44	9.45	4.08	2.59	16.9	3.56	6.39
	25	0.91	7.12	2.84	1.94	14.6	1.95	5.61	1.48	9.51	4.23	2.69	16.9	3.60	6.19
	26	0.80	7.08	2.79	1.93	13.9	3.05	5.85	1.38	8.96	4.00	2.57	15.0	2.38	5.98
	27	0.90	7.11	2.77	1.95	15.8	3.35	6.61	1.22	8.83	4.06	2.64	16.7	2.46	5.68
	28	0.94	7.14	2.75	2.01	15.3	2.52	6.28	1.27	9.23	4.06	2.58	17.5	3.39	6.25

Table S2. Values from the seven common phthalates measured in CHEAR QC pools A and B from 5 CHEAR studies. Batch numbers are provided per study and overall. All values are reported in ng/mL.

		Pool													
		A							B						
Study #	Run order	MBZP	MECPP	MEHHP	MEOHP	MEP	MIBP	MNBP	MBZP	MECPP	MEHHP	MEOHP	MEP	MIBP	MNBP
	29	0.97	7.00	2.73	1.93	15.3	2.60	5.46	1.48	8.85	4.05	2.60	17.3	3.43	6.53
3	30	0.84	3.57	2.20	2.24	13.2	3.69	5.98	1.49	6.11	2.70	3.15	12.2	4.69	7.69
	31	1.05	4.73	2.21	2.67	11.3	3.95	8.25	1.56	5.42	2.93	3.20	12.1	4.62	7.76
	32	0.62	3.15	1.79	1.74	15.0	3.63	7.48	1.50	4.60	3.10	2.86	11.6	3.92	8.07
	33	0.76	3.42	2.15	1.84	12.1	3.00	7.69	1.54	4.17	3.88	3.47	12.9	4.25	8.12
	34	1.35	3.83	2.13	2.75	13.5	4.65	8.90	1.37	6.10	2.83	3.36	14.9	4.70	8.00
	35	1.00	4.28	2.30	2.94	13.5	4.06	8.23	1.97	5.21	3.25	3.55	14.5	5.12	9.15
	36	1.23	4.37	2.58	3.13	12.9	4.44	8.98	1.71	5.18	3.51	3.32	14.8	5.52	8.93
4	37	1.25	4.50	2.26	2.42	13.6	3.55	8.23	1.66	6.16	3.10	3.20	15.4	4.99	8.86
	38	1.02	4.19	2.64	2.79	12.4	4.10	6.13	1.51	6.29	3.31	3.02	15.0	4.48	10.10
	39	0.94	3.93	2.20	2.84	14.4	3.71	8.40	1.50	4.84	3.64	3.49	15.7	4.04	8.24
	40	0.94	3.78	2.39	2.53	14.6	3.66	9.10	1.50	5.44	3.87	2.94	14.3	5.17	8.85
	41	0.90	3.97	2.05	2.27	15.7	4.43	8.78	1.71	6.33	3.71	3.47	15.6	4.68	8.40
	42	1.04	4.28	2.02	2.48	15.0	4.25	8.41	1.48	5.36	3.58	2.59	15.1	4.58	8.47
	43	0.90	4.32	2.51	2.55	13.1	4.62	8.32	1.32	6.61	3.87	3.04	14.7	5.35	8.62
	44	0.90	4.44	2.60	2.75	15.9	4.37	8.70	1.29	6.25	3.64	3.11	13.6	3.94	9.37
	45	1.05	4.99	2.29	2.46	15.3	4.89	8.72	1.73	6.65	3.61	3.00	15.9	5.47	10.20
	46	1.21	4.39	2.58	2.22	15.2	4.45	9.43	1.81	6.51	3.61	4.11	16.4	5.35	9.37
	47	1.05	4.13	2.23	2.68	13.8	4.04	8.51	1.39	6.01	3.39	3.47	15.3	4.36	8.08
	48	0.82	4.59	1.99	2.10	12.9	3.31	8.30	1.14	5.53	3.91	3.65	14.5	5.24	8.73
	49	1.08	3.72	2.20	1.92	10.8	4.51	8.34	1.67	5.18	3.94	2.75	15.0	5.30	10.10
	50	1.02	4.32	2.53	2.49	14.0	4.40	9.16	1.76	5.02	3.58	3.08	14.7	4.22	9.53
	51	0.99	4.93	2.23	2.86	15.2	4.83	8.66	1.49	5.42	3.77	3.37	12.0	5.59	9.01
	52	0.90	4.14	2.54	2.34	13.9	4.98	8.34	1.50	5.85	3.28	3.73	14.7	4.56	7.96
	53	1.16	4.38	2.39	3.10	12.0	5.12	5.92	1.33	6.38	3.67	3.17	14.0	4.81	8.12
	54	1.07	4.46	2.65	2.29	12.9	4.34	6.44	1.50	5.97	3.84	2.77	14.2	4.69	6.87
	55	1.11	4.74	2.16	2.74	14.6	4.88	8.86	1.48	6.37	3.70	3.33	14.6	5.78	9.95
	56	1.22	4.99	2.39	2.25	13.3	3.71	8.70	1.69	4.95	3.47	3.00	13.5	5.07	9.63

Table S2. Values from the seven common phthalates measured in CHEAR QC pools A and B from 5 CHEAR studies. Batch numbers are provided per study and overall. All values are reported in ng/mL.

		Pool													
		A							B						
Study #	Run order	MBZP	MECPP	MEHHP	MEOHP	MEP	MIBP	MNBP	MBZP	MECPP	MEHHP	MEOHP	MEP	MIBP	MNBP
4	57	0.81	3.32	2.10	2.29	14.1	4.13	8.64	1.45	5.97	3.35	3.22	14.0	5.53	8.99
	58	1.01	3.99	2.60	2.58	11.5	4.25	7.25	1.46	5.85	3.23	2.98	14.8	5.56	9.05
	59	0.73	4.38	2.36	2.13	12.0	4.77	7.90	1.45	6.45	3.35	2.42	12.4	5.80	9.69
	60	1.04	3.44	2.75	2.28	13.9	4.49	8.38	1.24	6.25	3.67	2.79	13.4	4.46	8.67
	61	1.09	4.16	2.34	2.61	14.1	3.32	9.03	1.56	6.47	3.16	3.26	15.0	3.97	9.46
	62	0.87	4.42	2.03	2.14	13.8	4.05	8.33	1.37	6.27	3.74	3.36	15.8	3.92	9.33
	63	1.06	4.84	1.94	2.48	13.5	4.54	7.69	1.84	5.96	3.16	3.16	15.2	4.34	9.70
	64	1.10	4.79	2.74	3.09	12.8	4.46	7.67	1.42	5.77	3.16	2.91	14.3	4.61	7.39
	65	1.00	4.16	2.12	2.38	15.0	4.30	8.15	1.54	5.89	3.32	3.46	14.8	4.60	8.00
	66	1.07	4.07	2.69	2.31	13.5	4.12	8.66	1.80	6.45	3.41	3.34	13.5	5.62	7.84
	67	1.09	4.08	1.98	3.08	14.3	4.23	9.10	1.31	5.00	3.76	2.96	13.8	4.99	9.66
	68	0.98	4.22	2.62	2.21	14.0	5.06	8.42	1.63	5.90	3.24	3.46	14.8	5.76	7.42
	69	1.08	4.09	2.70	1.77	15.0	4.27	7.89	1.63	5.54	3.65	3.28	14.8	5.33	9.72
5	70	1.01	3.12	4.13	1.65	22.0	4.35	8.91	1.44	3.80	7.00	2.12	23.2	3.94	9.28
	71	1.00	2.99	4.00	1.54	21.8	5.06	9.48	0.99	3.63	7.52	2.15	22.5	4.25	9.78
	72	1.04	3.08	4.25	1.81	22.0	4.37	9.08	1.22	3.94	7.36	2.15	23.0	5.95	9.78
	73	0.97	2.54	2.77	1.38	21.7	4.05	9.38	1.22	2.67	4.66	1.69	23.0	6.99	9.18
	74	1.09	2.61	3.20	1.42	21.9	3.67	10.30	1.43	2.79	4.50	1.59	23.0	4.45	8.58
	75	0.86	2.63	2.78	1.40	22.0	5.20	10.30	1.36	2.68	4.67	1.67	22.7	6.68	9.18
	76	0.77	1.17	4.67	2.44	21.6	4.88	10.50	1.35	1.11	6.35	2.38	22.5	4.04	9.16
	77	0.89	1.26	4.96	2.08	21.8	4.48	9.67	1.52	1.23	6.57	2.65	22.7	3.96	9.17
	78	0.89	0.90	4.52	2.00	21.6	4.05	9.27	1.64	1.37	7.18	2.63	23.1	4.75	9.57
	79	0.63	1.66	4.38	1.44	21.6	4.01	9.39	1.48	2.02	6.68	2.26	22.6	5.75	9.69
	80	0.79	1.91	4.87	1.56	21.7	5.17	9.39	0.66	2.18	8.63	2.28	22.6	5.24	9.49
	81	0.98	1.62	3.95	1.49	22.1	5.42	8.39	1.06	2.35	8.84	1.76	22.5	5.51	9.99
	82	1.40	3.17	4.64	1.77	21.5	3.72	10.10	1.10	3.82	6.28	2.74	23.4	5.71	10.30
	83	1.18	3.12	4.68	1.48	21.9	3.71	9.32	1.33	3.68	6.38	2.07	23.3	5.52	9.52
	84	1.28	3.05	4.13	1.62	22.4	4.73	10.10	1.11	3.77	6.38	2.68	23.0	5.34	10.00

Table S2. Values from the seven common phthalates measured in CHEAR QC pools A and B from 5 CHEAR studies. Batch numbers are provided per study and overall. All values are reported in ng/mL.

		Pool													
		A							B						
Study #	Run order	MBZP	MECPP	MEHHP	MEOHP	MEP	MIBP	MNBP	MBZP	MECPP	MEHHP	MEOHP	MEP	MIBP	MNBP
5	85	0.61	2.90	3.63	1.24	21.9	3.93	9.83	0.52	3.53	5.23	2.03	23.0	5.20	11.10
	86	1.02	2.87	3.54	1.77	21.8	3.60	9.53	0.99	3.51	5.01	1.79	23.0	5.18	10.20
	87	1.32	2.90	3.51	1.31	21.8	4.60	11.10	1.33	3.48	5.73	2.14	22.9	5.82	10.90
	88	1.15	3.19	3.77	2.04	21.7	4.39	9.92	1.43	3.41	4.61	2.03	22.7	4.44	10.50
	89	1.33	2.93	3.30	1.90	21.6	3.94	9.72	1.05	3.28	5.11	1.99	22.8	4.90	10.10
	90	1.32	2.92	2.99	1.74	21.5	4.57	10.10	1.61	3.67	5.37	2.26	22.8	4.08	10.20
