# Supplementary material for: Evaluating inter-study variability in phthalate and trace element analyses within the Children’s Health Exposure Analysis Resource (CHEAR) using multivariate control charts
Source: J Expo Sci Environ Epidemiol. 2021 Feb 18;31(2):318–27. doi: 10.1038/s41370-021-00293-w (PMC7952263; doi:10.1038/s41370-021-00293-w)
Supplement: Supplementary file 3 — Table S3 [file 41370_2021_293_MOESM3_ESM.pdf]

Table S3. Values from the 7 common phthalates measured in CHEAR QC pools A and B were z-scored utilizing combined means and SD from all 5 studies combined. Highly negative to highly positive values indicated by color scale red to green, respectively.

		Pool													
		A							B						
Study #	Run order	zMBZP	zMECPP	zMEHHP	zMEOHP	zMEP	zMIBP	zMNBP	zMBZP	zMECPP	zMEHHP	zMEOHP	zMEP	zMIBP	zMNBP
1	1	-0.2	0.3	0	-0.7	-1.2	-0.1	-1.3	-0.5	0.1	-0.1	-0.7	-1.4	5.8	-0.1
	2	-1	0.2	-0.1	-0.9	-1.2	-1.1	-1.4	-0.5	0.2	0.1	-0.5	-1.5	-0.5	-0.9
	3	-1.4	0.2	-0.3	-0.9	-0.3	-0.9	-1.6	-0.6	0.1	0	-0.5	-0.8	-0.3	-1
	4	-1.5	0.2	-0.2	-0.9	-0.3	-3.6	-2.1	-1.2	0	-0.3	-1	-0.3	-0.5	-1.5
2	5	0.2	1.5	0.3	0.2	0	-0.2	-0.7	-0.2	1.1	-0.1	-0.5	-0.4	0.2	-1.1
	6	0.3	1.4	0.3	0	-0.3	-0.9	-0.8	-0.3	1.1	-0.2	-0.2	-0.4	-1.2	-1.1
	7	0.6	1.4	0.4	0.2	-0.1	-1.5	-1.1	-0.1	1.1	-0.3	-0.2	-0.4	-1	-1.1
	8	0.6	1.3	0	0.2	-0.1	-0.6	-1	0	1.1	-0.4	-0.3	-0.3	-1.4	-1.3
	9	-0.4	1.3	-0.4	-0.3	-0.4	-1	-1.1	-0.2	1.1	-0.2	-0.5	-0.4	-1.6	-1.4
	10	-0.2	1.3	-0.1	-0.1	-0.2	-1.5	-1.3	0	1.4	-0.5	-0.1	-0.5	-1.2	-1.4
	11	0	1.2	0	-0.3	-0.2	-0.5	-1.2	-0.2	1.4	-0.5	0.1	-0.2	-1	-1.3
	12	0	1.2	0.3	-0.4	-0.4	-2.1	-1.2	-0.2	1.2	-0.7	-0.5	-0.2	-1.3	-1.3
	13	-0.1	1.5	0.1	-0.3	0	-1.5	-1.1	0.2	1.1	0.1	-0.3	-0.1	-1.5	-1.5
	14	0.5	1.6	-0.1	0	0	-1.5	-1	0.1	1.1	0.2	-0.2	-0.2	-0.7	-1.5
	15	-0.1	1.5	-0.2	-0.2	-0.2	-1.2	-0.9	0.5	1.6	0.4	-0.2	0.1	-0.9	-1.4
	16	0.6	1.7	0.3	-0.1	0.1	-0.9	-0.5	-0.1	1.4	0.3	-0.1	0	0.3	-0.8
	17	0.4	1.6	0.5	-0.2	0.1	-0.8	-0.8	0	1.5	0.1	-0.3	0.1	0.2	-1.3
	18	0.2	1.4	0.1	-0.3	-0.4	-0.8	-1.2	0.1	1.5	0	-0.2	0.2	-0.9	-0.9
	19	-0.2	1.4	0.1	-0.4	-0.4	0	-1.3	-0.1	1.5	0.2	-0.2	0.3	-0.2	-1.1
	20	0.3	1.4	-0.2	-0.3	-0.4	-1.3	-1.3	-0.1	1.4	0.1	-0.2	0	-0.9	-1
	21	0.4	1.5	0.3	0	0	-0.7	-1.1	-0.1	1.4	-0.1	-0.3	-0.2	-1.1	-1.4
	22	0.4	1.6	0.4	0	-0.2	-1.1	-1	-0.2	1.4	-0.1	-0.2	-0.5	-0.7	-1.5
	23	-0.6	1.5	-0.2	-0.5	-0.3	-1.3	-1.3	0	1.5	-0.1	-0.3	0	-0.7	-1.1
	24	-0.9	1.3	-0.1	-0.5	-0.3	-1.2	-1.4	0.1	1.5	-0.2	-0.3	0	-0.9	-1.3
	25	-0.6	1.3	-0.1	-0.4	-0.4	-2.1	-1.5	0.3	1.5	-0.1	-0.1	0	-0.8	-1.4
	26	-1.3	1.3	-0.1	-0.5	-0.6	-0.9	-1.3	-0.1	1.3	-0.3	-0.4	-0.5	-1.9	-1.5
	27	-0.6	1.3	-0.2	-0.4	-0.1	-0.5	-0.8	-0.9	1.2	-0.2	-0.2	0	-1.8	-1.7
	28	-0.4	1.3	-0.2	-0.3	-0.2	-1.5	-1	-0.6	1.4	-0.2	-0.4	0.2	-1	-1.3
	29	-0.2	1.3	-0.2	-0.5	-0.2	-1.4	-1.6	0.3	1.2	-0.2	-0.3	0.1	-1	-1.2
	30	-1	-0.6	-0.9	0.2	-0.8	-0.1	-1.2	0.4	0	-1.3	0.8	-1.3	0.1	-0.4

Table S3. Values from the 7 common phthalates measured in CHEAR QC pools A and B were z-scored utilizing combined means and SD from all 5 studies combined. Highly negative to highly positive values indicated by color scale red to green, respectively.

		Pool													
		A							B						
Study #	Run order	zMBZP	zMECPP	zMEHHP	zMEOHP	zMEP	zMIBP	zMNBP	zMBZP	zMECPP	zMEHHP	zMEOHP	zMEP	zMIBP	zMNBP
3	31	0.3	0	-0.9	1.2	-1.4	0.1	0.3	0.7	-0.3	-1.1	0.9	-1.3	0	-0.3
	32	-2.4	-0.8	-1.5	-0.9	-0.3	-0.2	-0.2	0.4	-0.6	-1	0.2	-1.5	-0.6	-0.1
	33	-1.5	-0.7	-1	-0.7	-1.2	-0.9	-0.1	0.6	-0.8	-0.4	1.4	-1.1	-0.3	-0.1
	34	2.2	-0.5	-1	1.4	-0.7	1	0.7	-0.2	0	-1.2	1.2	-0.5	0.1	-0.2
	35	0	-0.2	-0.8	1.9	-0.7	0.3	0.2	2.5	-0.4	-0.9	1.6	-0.7	0.4	0.6
	36	1.5	-0.2	-0.4	2.3	-0.9	0.7	0.7	1.3	-0.4	-0.7	1.1	-0.6	0.8	0.4
4	37	1.6	-0.1	-0.9	0.7	-0.7	-0.3	0.2	1.1	0	-1	0.9	-0.4	0.3	0.4
	38	0.1	-0.3	-0.3	1.5	-1.1	0.3	-1.1	0.4	0.1	-0.8	0.5	-0.5	-0.1	1.2
	39	-0.4	-0.4	-0.9	1.6	-0.5	-0.1	0.3	0.4	-0.5	-0.6	1.4	-0.3	-0.5	0
	40	-0.4	-0.5	-0.7	0.9	-0.4	-0.2	0.8	0.4	-0.3	-0.4	0.4	-0.7	0.5	0.4
	41	-0.6	-0.4	-1.1	0.3	-0.1	0.7	0.6	1.3	0.1	-0.5	1.4	-0.4	0.1	0.1
	42	0.3	-0.2	-1.2	0.8	-0.3	0.5	0.4	0.3	-0.3	-0.6	-0.3	-0.5	0	0.1
	43	-0.6	-0.2	-0.5	1	-0.9	0.9	0.3	-0.4	0.2	-0.4	0.6	-0.6	0.6	0.2
	44	-0.7	-0.1	-0.4	1.4	0	0.6	0.5	-0.5	0.1	-0.6	0.7	-0.9	-0.6	0.7
	45	0.3	0.2	-0.8	0.8	-0.2	1.2	0.6	1.4	0.3	-0.6	0.5	-0.3	0.7	1.3
	46	1.3	-0.2	-0.4	0.2	-0.2	0.7	1	1.8	0.2	-0.6	2.7	-0.1	0.6	0.7
	47	0.3	-0.3	-0.9	1.3	-0.7	0.3	0.4	-0.1	0	-0.8	1.4	-0.4	-0.2	-0.1
	48	-1.2	-0.1	-1.2	-0.1	-0.9	-0.6	0.3	-1.2	-0.2	-0.3	1.8	-0.7	0.5	0.3
	49	0.5	-0.5	-0.9	-0.5	-1.5	0.8	0.3	1.2	-0.4	-0.3	0	-0.5	0.6	1.2
	50	0.1	-0.2	-0.5	0.8	-0.6	0.7	0.8	1.6	-0.5	-0.6	0.6	-0.6	-0.3	0.9
	51	-0.1	0.1	-0.9	1.7	-0.2	1.2	0.5	0.4	-0.3	-0.4	1.2	-1.4	0.8	0.5
	52	-0.6	-0.3	-0.5	0.5	-0.6	1.3	0.3	0.4	-0.1	-0.8	1.9	-0.6	0	-0.2
	53	1	-0.2	-0.7	2.2	-1.2	1.5	-1.3	-0.4	0.1	-0.5	0.8	-0.8	0.2	-0.1
	54	0.4	-0.1	-0.3	0.4	-0.9	0.6	-0.9	0.4	0	-0.4	0	-0.7	0.1	-0.9
	55	0.7	0	-1	1.4	-0.4	1.2	0.6	0.3	0.1	-0.5	1.1	-0.6	1	1.1
	56	1.4	0.2	-0.7	0.3	-0.8	-0.1	0.5	1.3	-0.5	-0.7	0.5	-0.9	0.4	0.9
	57	-1.2	-0.8	-1.1	0.4	-0.6	0.4	0.5	0.2	0	-0.8	0.9	-0.8	0.8	0.5
	58	0.1	-0.4	-0.4	1	-1.3	0.5	-0.4	0.2	-0.1	-0.9	0.4	-0.6	0.8	0.5
	59	-1.7	-0.2	-0.7	0	-1.2	1.1	0	0.2	0.2	-0.8	-0.7	-1.2	1	1
	60	0.3	-0.7	-0.2	0.3	-0.6	0.8	0.3	-0.8	0.1	-0.5	0.1	-1	-0.1	0.3

Table S3. Values from the 7 common phthalates measured in CHEAR QC pools A and B were z-scored utilizing combined means and SD from all 5 studies combined. Highly negative to highly positive values indicated by color scale red to green, respectively.

		Pool													
		A							B						
Study #	Run order	zMBZP	zMECPP	zMEHHP	zMEOHP	zMEP	zMIBP	zMNBP	zMBZP	zMECPP	zMEHHP	zMEOHP	zMEP	zMIBP	zMNBP
	61	0.6	-0.3	-0.7	1.1	-0.6	-0.6	0.8	0.7	0.2	-0.9	1	-0.5	-0.5	0.8
	62	-0.8	-0.2	-1.2	0	-0.7	0.3	0.3	-0.2	0.1	-0.5	1.2	-0.3	-0.6	0.7
	63	0.4	0.1	-1.3	0.8	-0.7	0.8	-0.1	1.9	0	-0.9	0.8	-0.5	-0.2	1
	64	0.6	0	-0.2	2.2	-0.9	0.7	-0.1	0	-0.1	-0.9	0.3	-0.7	0	-0.6
4	65	0	-0.3	-1	0.6	-0.3	0.6	0.2	0.6	-0.1	-0.8	1.4	-0.6	0	-0.2
	66	0.4	-0.3	-0.3	0.4	-0.7	0.3	0.5	1.8	0.2	-0.7	1.1	-0.9	0.9	-0.3
	67	0.6	-0.3	-1.2	2.2	-0.5	0.5	0.8	-0.5	-0.5	-0.5	0.4	-0.9	0.3	0.9
	68	-0.1	-0.3	-0.4	0.2	-0.6	1.4	0.4	1	-0.1	-0.9	1.4	-0.6	1	-0.6
	69	0.5	-0.3	-0.3	-0.8	-0.3	0.5	0	1	-0.2	-0.5	1	-0.6	0.6	1
	70	0.1	-0.9	1.7	-1.1	1.8	0.6	0.7	0.1	-1	2.1	-1.3	1.8	-0.6	0.7
	71	0	-0.9	1.5	-1.4	1.7	1.4	1	-1.9	-1.1	2.5	-1.2	1.6	-0.3	1
	72	0.3	-0.9	1.8	-0.7	1.8	0.6	0.8	-0.9	-0.9	2.4	-1.2	1.7	1.1	1
	73	-0.2	-1.2	-0.2	-1.7	1.7	0.3	1	-0.9	-1.5	0.3	-2.1	1.7	2	0.6
	74	0.6	-1.1	0.4	-1.6	1.7	-0.2	1.6	0.1	-1.4	0.1	-2.3	1.7	-0.1	0.2
	75	-0.9	-1.1	-0.1	-1.7	1.8	1.6	1.6	-0.2	-1.5	0.3	-2.1	1.6	1.8	0.6
	76	-1.5	-1.9	2.4	0.7	1.6	1.2	1.7	-0.3	-2.2	1.6	-0.7	1.6	-0.5	0.6
	77	-0.7	-1.9	2.8	-0.1	1.7	0.8	1.2	0.5	-2.1	1.8	-0.2	1.6	-0.5	0.6
	78	-0.7	-2.1	2.2	-0.3	1.6	0.3	0.9	1	-2.1	2.3	-0.3	1.7	0.1	0.9
5	79	-2.4	-1.7	2	-1.6	1.6	0.2	1	0.3	-1.8	1.9	-1	1.6	1	1
	80	-1.4	-1.5	2.7	-1.3	1.7	1.6	1	-3.4	-1.7	3.4	-0.9	1.6	0.5	0.8
	81	-0.2	-1.7	1.4	-1.5	1.8	1.8	0.3	-1.6	-1.6	3.6	-2	1.6	0.8	1.2
	82	2.5	-0.8	2.4	-0.8	1.6	-0.1	1.4	-1.4	-1	1.5	0	1.8	0.9	1.4
	83	1.1	-0.9	2.4	-1.5	1.7	-0.1	0.9	-0.4	-1	1.6	-1.4	1.8	0.8	0.8
	84	1.8	-0.9	1.7	-1.2	1.9	1	1.4	-1.4	-1	1.6	-0.2	1.7	0.6	1.2
	85	-2.5	-1	1	-2	1.7	0.1	1.3	-4	-1.1	0.7	-1.4	1.7	0.5	1.9
	86	0.1	-1	0.9	-0.8	1.7	-0.3	1.1	-1.9	-1.1	0.5	-1.9	1.7	0.5	1.3
	87	2	-1	0.8	-1.9	1.7	0.9	2.1	-0.4	-1.1	1.1	-1.2	1.7	1	1.8
	88	1	-0.8	1.2	-0.2	1.7	0.7	1.3	0.1	-1.2	0.2	-1.4	1.6	-0.1	1.5
	89	2.1	-1	0.6	-0.5	1.6	0.1	1.2	-1.6	-1.2	0.6	-1.5	1.7	0.3	1.2
	90	2	-1	0.1	-0.9	1.6	0.9	1.4	0.9	-1	0.8	-1	1.7	-0.4	1.3
